# Reactivation of cytomegalovirus and bloodstream infection and its impact on early survival after allogeneic haematopoietic stem cell transplantation: a multicentre retrospective study

**DOI:** 10.3389/fmicb.2024.1405652

**Published:** 2024-06-19

**Authors:** Jinhua Ren, Jingjing Xu, Jiaqi Sun, Xueqiong Wu, Xiaozhu Yang, Chengjun Nie, Lingqiong Lan, Yanling Zeng, Xiaoyun Zheng, Jing Li, Qiaoxian Lin, Jianda Hu, Ting Yang

**Affiliations:** ^1^Department of Hematology, National Regional Medical Center, Binhai Campus of the First Affiliated Hospital, Fujian Medical University, Fuzhou, China; ^2^Department of Hematology, The First Affiliated Hospital, Fujian Medical University, Fuzhou, China; ^3^Institute of Precision Medicine, Fujian Medical University, Fuzhou, China; ^4^Department of Hematology, Fujian Institute of Hematology, Fujian Provincial Key Laboratory of Hematology, Fujian Medical University Union Hospital, Fuzhou, China; ^5^Department of Hematology, Ningde Hospital Affiliated to Ningde Normal University, Ningde, China; ^6^Department of Hematology, The Second Hospital of Longyan, Longyan, China; ^7^Department of Hematology, Affiliated Nanping First Hospital of Fujian Medical University, Nanping, China; ^8^The Second Affiliated Hospital, Fujian Medical University, Quanzhou, China

**Keywords:** allogeneic haematopoietic stem cell transplantation, cytomegalovirus reactivation, bloodstream infection, haematological disease, acute myeloid leukaemia

## Abstract

Cytomegalovirus reactivation (CMVr) and bloodstream infections (BSI) are the most common infectious complications in patients after allogeneic haematopoietic stem cell transplantation (allo-HSCT). Both are associated with great high morbidity whilst the BSI is the leading cause of mortality. This retrospective study evaluated the incidence of CMVr and BSI, identified associated risk factors, assessed their impact on survival in allo-HSCT recipients during the first 100 days after transplantation. The study comprised 500 allo-HSCT recipients who were CMV DNA-negative and CMV IgG-positive before allo-HSCT. Amongst them, 400 developed CMVr and 75 experienced BSI within 100 days after allo-HSCT. Multivariate regression revealed that graft failure and acute graft-versus-host disease were significant risk factors for poor prognosis, whereas CMVr or BSI alone were not. Amongst all 500 patients, 56 (14%) developed both CMVr and BSI in the 100 days after HSCT, showing significantly reduced 6-month overall survival (*p* = 0.003) and long-term survival (*p* = 0.002). Specifically, in the initial post-transplant phase (within 60 days), BSI significantly elevate mortality risk, However, patients who survive BSI during this critical period subsequently experience a lower mortality risk. Nevertheless, the presence of CMVr in patients with BSI considerably diminishes their long-term survival prospects. This study provides real-world data on the impact of CMVr and BSI following transplantation on survival, particularly in regions such as China, where the prevalence of CMV IgG-positivity is high. The findings underscore the necessity for devising and executing focused prevention and early management strategies for CMVr and BSI to enhance outcomes for allo-HSCT recipients.

## 1 Introduction

Allogeneic haematopoietic stem cell transplantation (allo-HSCT) is an effective and intensive treatment for many haematological diseases, especially acute leukaemia. Nevertheless, post-allo-HSCT infection contributes to early mortality. The Center for International Blood and Marrow Transplant Research (CIBMTR) (2024) database shows that infection accounts for 19% and 28% of mortality within 100 days post-transplantation in matched-related and haploidentical HSCT amongst recipients aged over 18, respectively. Amongst various complications, bloodstream infection (BSI) and cytomegalovirus reactivation (CMVr) are the two most common infections after allo-HSCT (Cho et al., 2019; Ljungman et al., 2019).

Several factors can influence the development of BSI, including immunodeficiency resulting from long-term myelosuppression and immunosuppression after allo-HSCT (Girmenia et al., 2017; Yan et al., 2018). Furthermore, CMV infection has been associated with myelosuppression. Various sources, such as *in vitro* studies, statistical analyses of clinical studies, patient case reports, and studies in murine models (Randolph-Habecker et al., 2002) collectively provide evidence of a reciprocal relationship between CMVr and BSI after allo-HSCT.

Infections following allo-HSCT are not typically observed in clinical practice as isolated events but frequently manifest in combination with other infections. Several studies have extensively explored the characteristics of BSI or CMVr after HSCT; some of these studies have indicated an association between CMVr and invasive fungal infections, Epstein–Barr virus reactivation, or hepatitis B virus reactivation (Liu et al., 2012; Zhou et al., 2020; Ding et al., 2021; Li et al., 2022; Zhang et al., 2022). Nevertheless, a limited number of studies have delved into the relationship between CMVr and BSI shortly after HSCT. This study aimed to delineate the incidence of CMVr and BSI amongst patients who underwent allo-HSCT in the southeastern coastal region of China from March 2013 to May 2022. Additionally, it sought to elucidate the effects of BSI and CMVr occurring within the first 100 days after transplantation on recipient clinical outcomes.

## 2 Materials and methods

### 2.1 Study design and patient selection

This study constituted a multicentre, retrospective analysis of a patient cohort presenting with haematological disorders who underwent allo-HSCT in four hospitals across the Fujian Province of China over a 9-year period (March 2013 to May 2022). A total of 500 patients who underwent their first allo-HSCT were included, and their medical records were retrospectively reviewed. All the included patients, children and adults, were consecutive. Before participation in the study, all patients and donors provided informed consent. The research was conducted in accordance with and approved by the ethical standards of the local institutional review board (Medical Ethics Committee of Fujian Medical University Union Hospital, 2022KY167) and the Declaration of Helsinki.

### 2.2 Transplantation procedures

Patients diagnosed with acute myeloid leukaemia, acute lymphoblastic leukaemia, and myelodysplastic syndrome (MDS) were treated with a standard myeloablative conditioning regimen. This regimen consisted of fludarabine (25 mg/m^2^/day intravenously on days −10 to −6), cytarabine (2 g/m^2^/day intravenously on days −10 to −6), busulfan (0.8 mg/kg/q6hr intravenously on days −6 to −4), cyclophosphamide (1.8 g/m^2^/day intravenously on days −3 to −2), semustine (250 mg/m^2^ orally on day −1), and anti-thymocyte globulin (ATG; 7.5–10 mg/kg intravenously on days −5 to −2). Patients diagnosed with aplastic anaemia received fludarabine (30 mg/m^2^/day intravenously on days −5 to −2), cyclophosphamide (30 mg/kg/day intravenously on days −5 to −2), and ATG (2.5 mg/kg/day intravenously on days −4 to −2). All patients received a graft-versus-host disease (GVHD) prophylaxis consisting of cyclosporine, mycophenolate mofetil, and short-term methotrexate.

### 2.3 CMV therapy and monitoring

Testing for CMV was performed in plasma samples using real-time quantitative polymerase chain reaction with a CMV nucleic acid quantitative detection kit (DA-D061; Da An Gene Co., Ltd., Guangzhou, China). All centres employ the same CMV testing kit, along with identical detection procedures and instruments. CMV screening started from the first day after transplantation, twice a Week. All patients received prophylactic acyclovir (5 mg/kg, every 8 h) from days 1 to 100. CMVr was defined as a viral load of >500 copies/mL on two consecutive readings 5 days apart (Mo et al., 2022). The time of CMVr refers to the duration from allo-HSCT to the occurrence of CMVr. Preemptive therapy with either intravenous foscarnet (90 mg/kg/day) or intravenous ganciclovir (5 mg/kg, twice daily) was initiated upon confirmation, if the CMV viral load continues to increase after 2 weeks of ganciclovir continuous treatment, we switch to valganciclovir (900 mg, twice daily) or foscarnet (120 mg/kg/day) for treatment, therapy was continued until CMV DNA was no longer detected in two consecutive tests.

### 2.4 Definition and management of BSI

BSI was defined as an infection resulting from a bacterial or fungal pathogen isolated from at least one blood culture. In the case of coagulase-negative S*taphylococcus* infection or common skin contaminants, a minimum of two positive blood cultures were required (Frère et al., 2004; Cappellano et al., 2007). Blood cultures were obtained when signs of infection, typically manifesting as fever (oral temperature of ≥38°C), were observed. All suspected infected catheters were aseptically removed, and catheter tips were cultured to confirm catheter-related BSI. Prophylaxis for fungal infection included primary prophylaxis for patients without a prior history of invasive fungal infection (IFD) and secondary prophylaxis for patients with a prior history of IFD. Primary prophylaxis consisted of oral posaconazole or itraconazole from day −10 to day +100 after transplant. Secondary prophylaxis involves the use of antifungal medications that have been effective in the patient’s previous treatment from −10 days to +100 days post-transplantation. No antibacterial prophylaxis was conducted before transplantation. All patients with febrile neutropenia were treated promptly with empiric intravenous broad-spectrum antibiotics until the results of the blood and catheter tip cultures were known, following current guidelines (Tomblyn et al., 2009; Averbuch et al., 2013a,b; Heinz et al., 2017).

### 2.5 Statistical analyses

The analysis of the data encompassed several variables aimed at identifying risk factors for CMV and BSI: age, sex, underlying diseases, disease status at transplantation, donor type, conditioning regimen, presence of acute GVHD (aGVHD), ATG dose and BSI. Kaplan–Meier survival analysis was used to calculate survival and the cumulative incidence of CMV infection and BSI. We performed landmark analyses for all endpoints by dividing the entire 3,400-day follow-up period into the first 60 days and subsequent 3,340 days. Differences between the two survival curves were estimated using a log-rank test. Multivariate Cox proportional hazards regression analysis was performed to identify prognostic factors for CMVr and BSI. Statistical significance was set at 0.05 for *p*-values. All statistical analyses were performed using R (4.3.0) and Prism 8 (Graphpad Software, La Jolla, CA, United States) software.

## 3 Results

### 3.1 Characteristics of patients undergoing allo-HSCT

Five patients with CMV DNA levels of >10^3^ copies/mL before allo-HSCT were excluded from further analysis. In this study, we enrolled 500 patients, all of whom, including both donors and recipients, were CMV IgG-positive before transplantation. The stem cells were all derived from bone marrow and peripheral blood; there were no patients who underwent umbilical cord blood stem cell transplantation. The cohort comprised 64.8% (*n* = 324) men and 35.2% (*n* = 176) women, with a median age of 26 (IQR 12–40) years. More than half of the patients were diagnosed with acute leukaemia (41.6% with acute myeloid leukaemia and 23% with acute lymphoblastic leukaemia). Other diseases included chronic myeloid leukaemia, severe aplastic anaemia, MDS, non-Hodgkin lymphoma, and congenital immune deficiency. A myeloablative conditioning regimen was administered to 77.2% (*n* = 386) of the patients, and an ATG dose of ≥7.5 mg/kg was administered to 89.6% (*n* = 448). Amongst the 500 patients, the types of HSCT donors included 355 (71%) haploidentical donors, 101 (20.2%) HLA-matched siblings, and 44 (8.8%) unrelated donors. Neutrophil engraftment was achieved in 97% (*n* = 485) of patients, and the median time to absolute neutrophil count recovery was 13 (IQR: 12–16) days. Platelet engraftment occurred in 92% (*n* = 460) of the patients, with a median time to platelet recovery of 15 (IQR: 12–19) days. The overall incidence of acute GVHD (grades I–IV) was 33.2% (*n* = 166), whereas that of severe acute GVHD (grades III–IV) was 18.8% (*n* = 94). The median follow-up time was 606 (IQR: 170–1,433) days. Table 1 summarises the characteristics of all participants.

**Table 1:** Baseline characteristics of patients receiving allogeneic haematopoietic stem cell transplantation (allo-HCST).

Characteristics	Total
Age, median (IQR)	26 (12, 40)
**Patient Sex, *n* (%)**	
Male	324 (64.8%)
Female	176 (35.2%)
**Disease, *n* (%)**	s
AML	208 (41.6%)
ALL	115 (23%)
MDS	32 (6.4%)
CML	25 (5%)
AA	79 (15.8%)
AITL	7(1.4%)
Lymphoma	17(3.4%)
Other	17(3.4%)
**Pretreatment status, *n* (%)**	
RIC	114 (22.8%)
MAC	386 (77.2%)
**ATG dose, *n* (%)**	
5 mg/kg	52 (10.4%)
7.5 mg/kg	196 (39.2%)
10 mg/kg	252 (50.4%)
**HLA-matching status, *n* (%)**	
Mismatch	355 (71%)
Match	101(20.2%)
Unrelated donor	44 (8.8%)
**Neutrophil engraftment, *n* (%)**	
Engraftment	485 (97%)
Failure	15 (3%)
Neutrophil count recovery (day), median (IQR)	13 (12–16)
**PLT engraftment, *n* (%)**	
Engraftment	460 (92%)
Failure	40 (8%)
PLT count recovery (day), median (IQR)	15 (12–19)
**aGVHD, *n* (%)**	
No aGVHD	334 (66.8%)
Grade I-II	72 (14.4%)
Grade III-IV	94 (18.8%)
**CMVr**	
No CMVr	100 (20%)
CMVr	400 (80%)
**BSI**	
No BSI	425(81.2%)
BSI	75(18.8%)
CMVr Time (day)*, median (IQR)	32 (24, 41)
Follow-up time (day), median (IQR)	606 (170, 1,433)

IQR, Interquartile Range; AML, Acute Myeloid Leukaemia; ALL, Acute Lymphoblastic Leukaemia; MDS, Myelodysplastic Syndromes; CML, Chronic Myeloid Leukaemia; AA, Aplastic Anaemia; AITL, Angioimmunoblastic T-cell Lymphoma; PLT, Platelets; aGVHD, acute Graft-Versus-Host Disease; RIC, Reduced-Intensity Conditioning; MAC, Myeloablative conditioning; CMVr, Cytomegalovirus reactivation; CMVr Time*, The duration from haematopoietic stem cell transplantation to the occurrence of CMVr; CMVr duration time*, The time from CMV reactivation to CMV clearance.

### 3.2 CMV reactivation and BSI development in allo-HSCT

Of the 500 patients who underwent allo-HSCT, 400 (80%) experienced CMVr within the first 100 days after allo-HSCT. The median time to CMVr occurrence was 32 (IQR: 24–41) days. The median duration of CMV viraemia was 31 (IQR: 24–41) days (Table 1). Within 100 days after HSCT, CMV was cleared in 77.8% (*n* = 311) of the patients, with a median time of 67 (IQR: 53–90) days and the median time of duration of CMVr was 42 (IQR: 27–77) days (Table 2). Amongst all 500 patients, 75 (18.8%) developed BSI, of whom 56 experienced CMVr within the first 100 days. A total of 70 different strains were detected in these patients. Blood culture specimens from 43 patients showed the presence of a single pathogen, whilst 12 showed two pathogens, and one showed three pathogens. The detection rate of Gram-negative bacteria was 56%, with predominant pathogens being *Pseudomonas aeruginosa* and *Escherichia coli*. Gram-positive bacteria were detected in 34.7% of cases, with Staphylococcus and Streptococcus being the main species. Additionally, fungi were detected in 6 cases, as shown in Supplementary Table 1. Of the 75 patients with BSI, 35 (46.7%) were found to have multidrug-resistant (MDR) bacterial infection. The predominant MDR bacteria were mainly *Escherichia coli* and *Klebsiella pneumoniae*, with the Extended spectrum β-lactamase-producing (ESBL+) strains had the highest resistance rate 16 (21.3%), as showed in Supplementary Table 2.

**Table 2:** Baseline characteristics of all patients in the cytomegalovirus reactivation (CMVr) and no-CMVr groups.

Characteristics	CMVr	No CMVr	*p*-value
	*N* = 400	*N* = 100	
Age, median (IQR)	26 (13, 40)	25 (11, 39.25)	0.851
Disease, *n* (%)			0.056
AA	64 (16%)	15 (15%)	
AML	165 (41.2%)	43 (43%)	
MDS	24 (6%)	8 (8%)	
Lymphoma	17 (4.2%)	0 (0%)	
CML	15 (3.8%)	10 (10%)	
ALL	94 (23.5%)	21 (21%)	
Other	16 (4%)	1 (1%)	
AITL	5 (1.2%)	2 (2%)	
Disease status, *n* (%)			0.107
Not CR	200 (50%)	59 (59%)	
CR	200 (50%)	41 (41%)	
Donor Sex, *n* (%)			0.135
Male	264 (66%)	58 (58%)	
Female	136 (34%)	42 (42%)	
HLA-matching status, *n* (%)			0.079
Unrelated donor	39 (9.8%)	5 (5%)	
Mismatch	287 (71.8%)	68 (68%)	
Match	74 (18.5%)	27 (27%)	
MNC, median (IQR)	7.955 (6.4, 11.08)	7.8 (5.98, 11.592)	0.663
CD34, median (IQR)	4.295 (2.8975, 7.44)	3.75 (2.7, 7.795)	0.574
Neutrophil engraftment, *n* (%)			<0.001
Engraftment	398 (99.5%)	87 (87%)	
Failure	2 (0.5%)	13 (13%)	
Neutrophil engraftment time, median (IQR)	13 (12, 16)	13 (12, 16.5)	0.397
PLT engraftment, *n* (%)			<0.001
Engraftment	379 (94.8%)	81 (81%)	
Failure	21 (5.2%)	19 (19%)	
PLT engraftment time, median (IQR)	15 (12, 19)	14 (12, 18)	0.122
aGVHD, *n* (%)			0.574
Grade I–II	55 (13.8%)	17 (17%)	
Grade III–IV	78 (19.5%)	16 (16%)	
No GVHD	267 (66.8%)	67 (67%)	
Pretreatment, *n* (%)			0.070
RIC	98 (24.5%)	16 (16%)	
MAC	302 (75.5%)	84 (84%)	
ATG dose, *n* (%)			0.004
7.5 mg/kg	216 (54%)	36 (36%)	
10 mg/kg	143 (35.8%)	53 (53%)	
5 mg/kg	41 (10.2%)	11 (11%)	
BSI, *n* (%)			0.210
BSI	56 (14%)	19 (19%)	
No BSI	344 (86%)	81 (81%)	
CMV cleared status*, n (%)			
Clear	311 (77.8%)		
Unclear	89 (22.2%)		
Time to clear CMV (day), median (IQR)	67 (53, 90)		
Duration of CMVr (day), median (IQR)	42 (27, 77)		
Follow up time (day), median (IQR)	633.5 (184.75, 1376.5)	388 (76.75, 2,148)	0.306

CMVr, Cytomegalovirus Reactivation; BSI, Bloodstream Infection; IQR, Interquartile Range; AML, Acute Myeloid Leukaemia; ALL, Acute Lymphoblastic Leukaemia; MDS, Myelodysplastic Syndromes; CML, Chronic Myeloid Leukaemia; AA, Aplastic Anaemia; AITL, Angioimmunoblastic T-cell Lymphoma; Not CR, partial response, stable disease (SD), and progressive disease; CR, Complete Response; MNC, Mononuclear Cell; PLT, Platelets; aGVHD, acute Graft-Versus-Host Disease; MAC, Myeloablative Conditioning; RIC, Reduced-Intensity Conditioning; ATG dose, Anti-Thymocyte Globulin dose; CMV Cleared status*, CMV cleared within 100 days after haematopoietic stem cell transplantation.

### 3.3 Risk factors in patients with HSCT

Cox regression analysis of survival in 500 patients undergoing allo-HSCT was used to identify significant factors related to OS. Neutrophil (*p* < 0.001; HR 25.19 [95% CI 10.135–62.585]) and platelet engraftment failure (*p* < 0.001; HR 3.505 [95% CI 1.990–6.175]), as well as grade I–II aGVHD (*p* = 0.022; HR: 1.841 [95% CI 1.091–3.107]) and grade III–IV aGVHD (*p* < 0.001; HR: 3.399 [95% CI 2.221–5.202]), were significant predictors of OS. Notably, CMVr was not identified as a risk factor for OS in the competing risk model (*p* > 0.05). Table 3 summarises the hazards for OS after allo-HSCT.

**Table 3:** Cox proportional hazard regression analysis for overall survival (OS) after allogeneic haematopoietic stem cell transplantation (allo-HCST).

Characteristics	Total (*N*)	Univariate analysis	*p*-value	Multivariate analysis	*p*-value
		Hazard ratio (95% CI)		Hazard ratio (95% CI)	
Patient age	500		0.016		
<18	137	Reference		Reference	
≥18	363	1.651 (1.075–2.537)	0.022	1.447 (0.915–2.288)	0.114
Patient sex	500		0.829		
Male	324	Reference			
Female	176	0.961 (0.670–1.379)	0.830		
Disease	500		0.027		
Acute leukaemia	355	Reference		Reference	
Non-acute leukaemia	145	0.636 (0.419–0.966)	0.034	0.838 (0.439–1.598)	0.592
Disease status	500		0.104		
CR	241	Reference			
Not CR	259	1.331 (0.941–1.881)	0.106		
Donor sex	500		0.778		
Male	322	Reference			
Female	178	0.950 (0.664–1.360)	0.779		
Donor age	500		0.070		
<18	40	Reference		Reference	
≥18	460	1.974 (0.870–4.479)	0.104	1.003 (0.429–2.347)	0.995
HLA-match	500		0.095		
Unrelated donor	44	Reference		Reference	
Match	101	0.691 (0.326–1.463)	0.334	0.660 (0.299–1.454)	0.303
Mismatch	355	1.163 (0.624–2.167)	0.634	0.556 (0.281–1.101)	0.092
Donor-recipient blood type	500		0.361		
Match	319	Reference			
Major mismatched	59	1.450 (0.897–2.343)	0.129		
Minor mismatched	90	0.845 (0.518–1.378)	0.500		
Minor and Major mismatched	32	1.145 (0.575–2.279)	0.701		
MNC	500	0.973 (0.935–1.012)	0.166		
CD34	500	0.995 (0.961–1.031)	0.801		
Neutrophil engraftment	500		**<0.001**		
Engraftment	485	Reference		Reference	
Failure	15	51.597 (27.663–96.241)	**<0.001**	25.186 (10.135–62.585)	<0.001
PLT engraftment	500		**<0.001**		
Engraftment	460	Reference		Reference	
Failure	40	11.879 (7.983–17.676)	**<0.001**	3.505 (1.990–6.175)	**<0.001**
aGVHD	500		**<0.001**		
No aGVHD	330	Reference		Reference	
aGVHD I–II	77	1.688 (1.044–2.730)	0.033	1.841 (1.091–3.107)	**0.022**
aGVHD III–IV	93	3.423 (2.339–5.008)	**<0.001**	3.399 (2.221–5.202)	**<0.001**
BSI	500		**<0.001**		
No-BSI	425	Reference		Reference	
BSI	75	2.957 (2.026–4.316)	**<0.001**	1.936 (0.974–3.847)	0.060
CMVr	500		0.001		
CMVr	400	Reference		Reference	
No-CMVr	100	1.914 (1.309–2.801)	**<0.001**	1.229 (0.528–2.860)	0.633
CMVr time	500	1.010 (1.005–1.016)	**<0.001**	1.004 (0.992–1.015)	0.519
Pretreatment	500		0.001		
RIC	114	Reference		Reference	
MAC	386	2.135 (1.282–3.554)	0.004	1.519 (0.659–3.503)	0.327
ATG dose	500		0.024		
5 mg/kg	52	Reference		Reference	
7.5 mg/kg	252	1.636 (0.783–3.422)	0.191	2.115 (0.879–5.085)	0.094
10 mg/kg	196	2.301 (1.103–4.803)	0.026	2.155 (0.924–5.025)	0.075

CMVr, Cytomegalovirus Reactivation; BSI, Bloodstream Infection; IQR, Interquartile Range; Not CR, partial response, stable disease (SD), and progressive disease; CR, Complete Response; MNC, Mononuclear Cell; PLT, Platelets; aGVHD, acute Graft-Versus-Host Disease; MAC, Myeloablative Conditioning; RIC, Reduced-Intensity Conditioning; ATG dose, Anti-Thymocyte Globulin dose.

**Figure 1:**
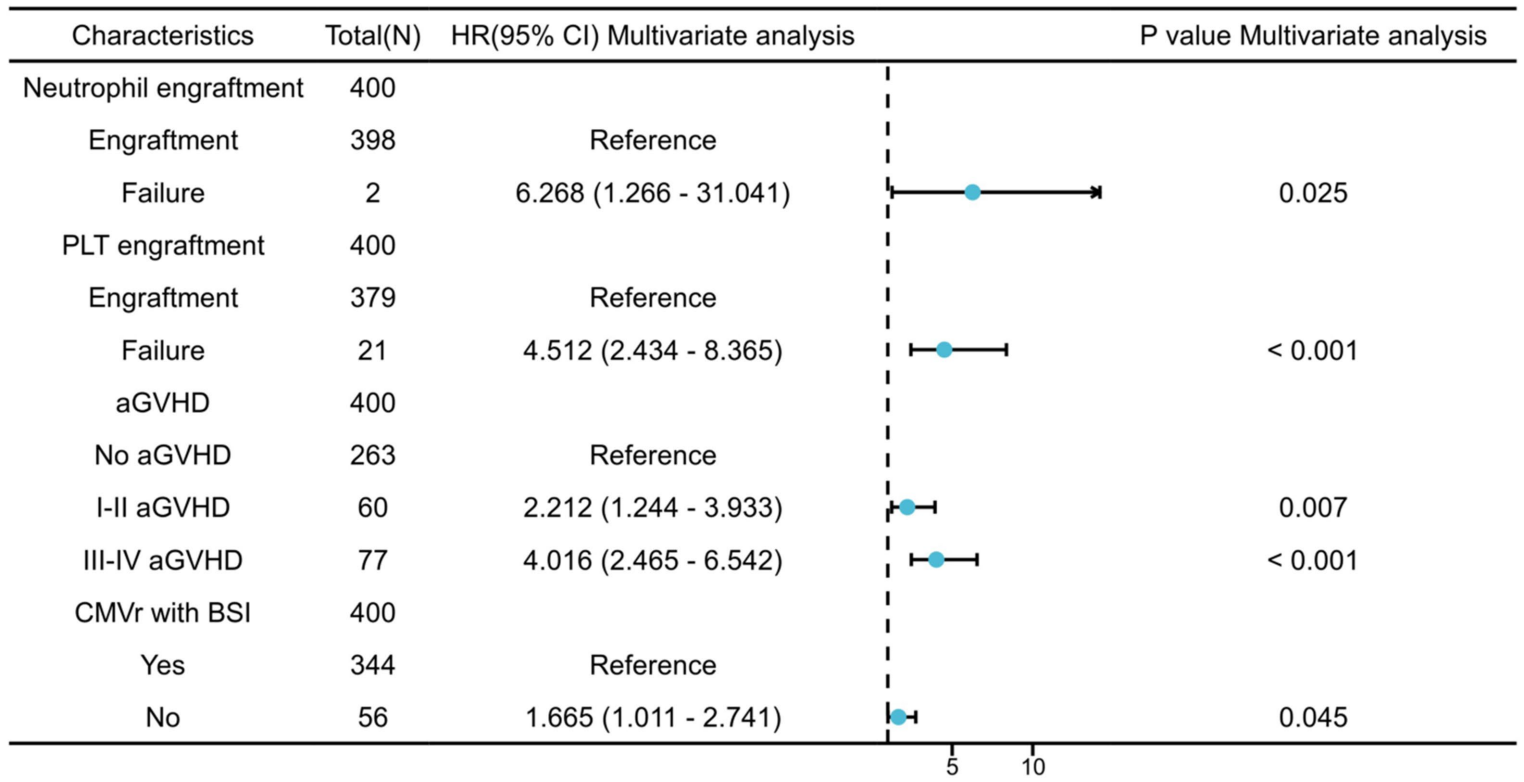
Risk factors for overall survival in patients with cytomegalovirus reactivation after allogeneic haematopoietic stem cell transplantation.

We further analysed the risk factors leading to mortality in patients with CMVr. Specifically, neutrophil engraftment failure (*p* = 0.025; hazard ratio (Heinz et al., 2017) 6.268 [95% confidence interval {CI} 1.266–31.041]) and platelet engraftment failure (*p* < 0.001; HR 4.512 [95% CI 2.434–8.365]), as well as grade I–II aGVHD (*p* = 0.007; HR 2.212 [95% CI 1.244–3.933]) and grade III–IV aGVHD (*p* < 0.001; HR 4.016 [95% CI 2.465–6.542]), were identified as significant risk factors for overall survival (OS). Additionally, CMVr with BSI (*p* = 0.045; HR 1.665 [95% CI 1.011–2.741]) was also a significant risk factor for OS in the CMVr patients. Figure 1 summarises the factors related to OS in CMVr.

### 3.4 Landmark analysis the impact of CMVr and BSI on post-transplantation survival

**Figure 2:**
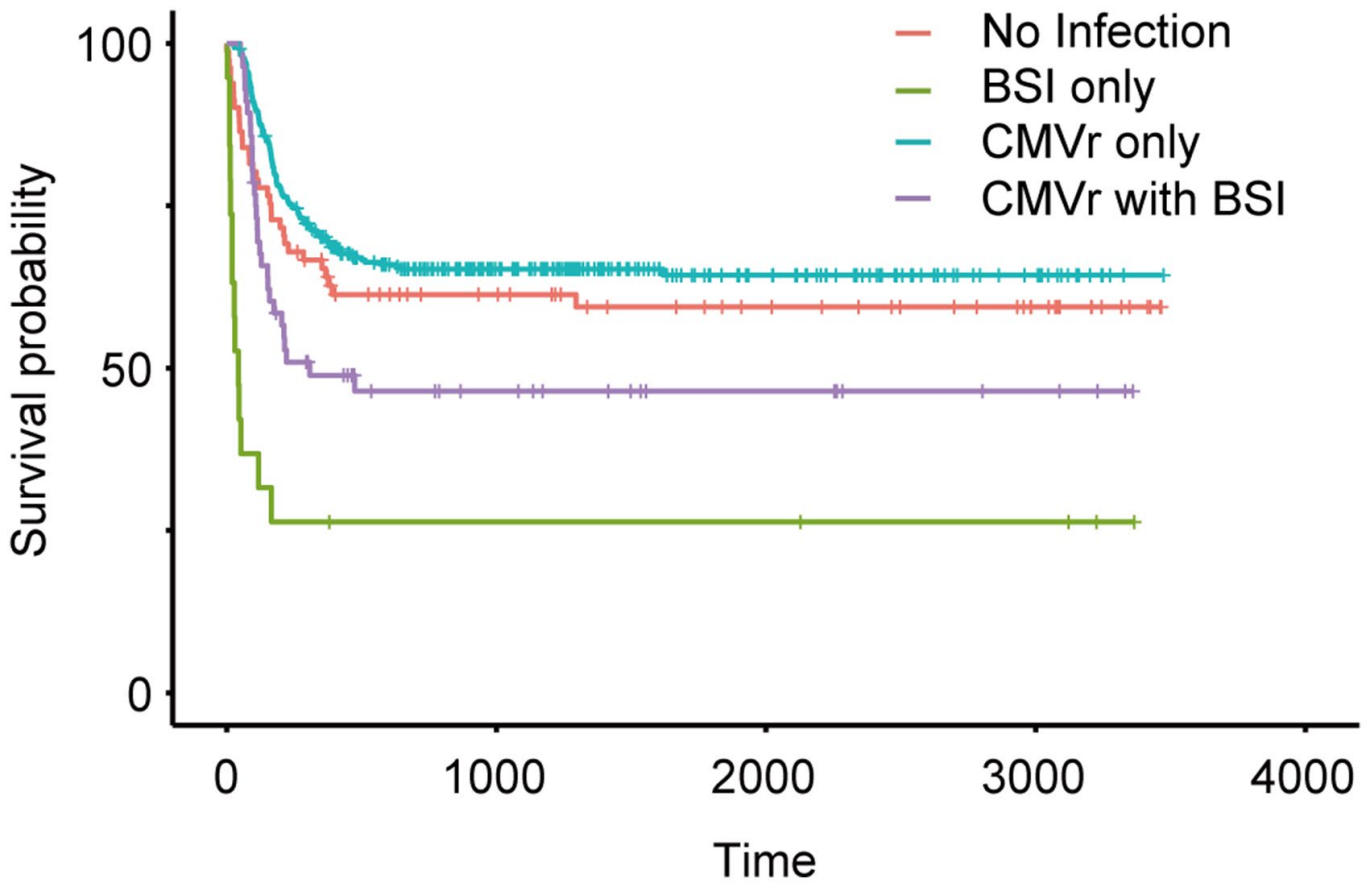
Kaplan–Meier analysis of long-term survival in all patients after allogeneic haematopoietic stem cell transplantation (allo-HCST).

To investigate the impact of BSI and CMVr on the survival of HSCT patients, we categorised all patients into four groups (based on infections within first 100 days after transplantation): (1) no infection; (2) BSI only; (3) CMVr only; (4) CMVr with BSI. Kaplan–Meier analysis of survival outcomes indicated that the CMVr-only group had the best survival, followed by the no-infection group and CMVr with BSI group. The BSI-only group demonstrated the lowest survival (Figure 2). Notably, survival curves demonstrated a crossover around 60 days. A significant early decline in survival was observed for the BSI-only group and CMVr with BSI group during the immediate post-transplantation period, which was not observed in other groups. It seems that the influence of BSI on OS ‘outshines’ the CMVr variable considerably and distorts the results.

**Figure 3:**
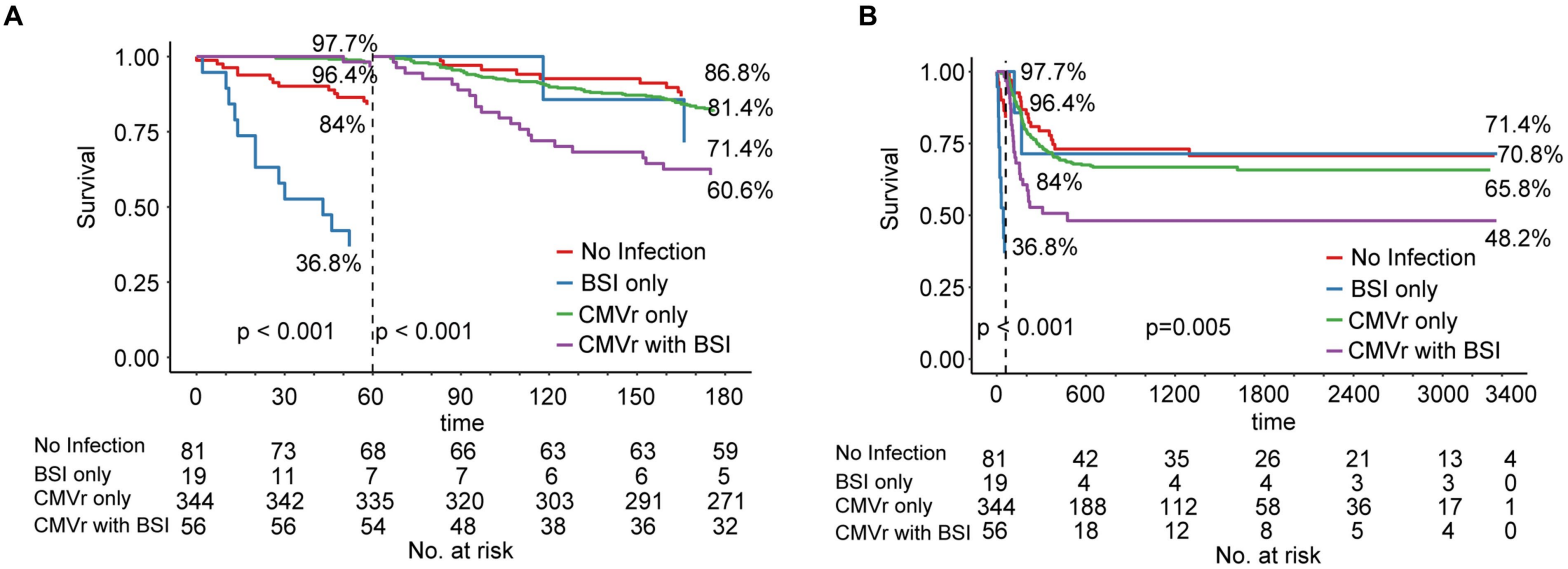
**(A)** Landmark analysis of overall survival for all patients within 180 days after allogeneic haematopoietic stem cell transplantation (allo-HCST); landmark point set at 60 days after transplantation; *p* < 0.001 before and after 60 days; **(B)** Landmark analysis of overall survival for all patients after allo-HSCT patients in long-term follow-up; landmark point set at 60 days after transplantation; before 60 days, *p* < 0.001; after 60 days, *p* = 0.005.

Due to changes in death risk of BSI and CMVr over different follow-up time intervals, we employed landmark analysis to separately analyse the data in different time periods, with 60 days post-transplantation as the landmark time point. During the first 6 months after transplantation, the OS for the CMVr with BSI group exhibited significant fluctuations, with a decrease from 96.45% before 60 days after transplantation to 60.6% thereafter. Notably, within the initial 60 days after transplantation, the BSI-only group demonstrated the lowest survival, with an OS of 36.8%. This improved to 71.4% after 60 days, whilst the OS for the CMVr with BSI group reduced to 60.6% (Figure 3A). After 60 days following transplantation, the OS for the BSI-only group remained at 71.4%, the OS for the CMVr-only group decreased to 65.8%, and the OS of the CMVr with BSI group showed a marked decline in survival to 48.2% (Figure 3B). This suggests that the risk of BSI-related mortality is high within the first 60 days post HSCT, but decreases significantly if the patient survives beyond this period. However, for patients with concurrent CMVr, the risk of mortality increases substantially after 60 days post-transplantation. Meanwhile, we attempted to use 30 days post-transplantation as the landmark time point, but the results did not reveal a turning time point of BSI survival (Supplementary Figures 1A,B).

An analysis of baseline data across the four groups indicated poor neutrophil and platelet engraftment in the BSI group. In the BSI-only group, neutrophil engraftment failure occurred in 26.3% of patients, and platelet engraftment failure occurred in 42.1% of patients, significantly higher than the rates in the CMVr-only group (0.6 and 4.1%, respectively) and the CMVr with BSI group (0% and 12.5%, respectively; characteristics summarised in Table 4). The elevated mortality rate in the BSI group during the initial post-transplantation period may be linked to this suboptimal engraftment.

**Table 4:** Baseline characteristics of patients in the no-infection, bloodstream infection (BSI)-only, cytomegalovirus reactivation (CMVr)-only, and CMVr with BSI groups.

Characteristics	No Infection	BSI-only	CMVr-only	CMVr with BSI	*p*-value
	*N* = 81	*N* = 19	*N* = 344	*N* = 56	
Age, median (IQR)	24 (10, 37)	33 (20, 49.5)	26 (12, 40)	26 (17.75, 37.5)	0.183
Patient Sex, *n* (%)					0.250
Male	59 (72.8%)	14 (73.7%)	218 (63.4%)	33 (58.9%)	
Female	22 (27.2%)	5 (26.3%)	126 (36.6%)	23 (41.1%)	
Disease, *n* (%)					0.326
AML	33 (40.7%)	10 (52.6%)	143 (41.6%)	22 (39.3%)	
ALL	17 (21%)	4 (21.1%)	79 (23%)	15 (26.8%)	
Other	1 (1.2%)	0 (0%)	14 (4.1%)	2 (3.6%)	
MDS	6 (7.4%)	2 (10.5%)	20 (5.8%)	4 (7.1%)	
AA	12 (14.8%)	3 (15.8%)	57 (16.6%)	7 (12.5%)	
CML	10 (12.3%)	0 (0%)	13 (3.8%)	2 (3.6%)	
Lymphoma	0 (0%)	0 (0%)	13 (3.8%)	4 (7.1%)	
AITL	2 (2.5%)	0 (0%)	5 (1.5%)	0 (0%)	
Disease status, *n* (%)					0.083
CR	37 (45.7%)	4 (21.1%)	170 (49.4%)	30 (53.6%)	
Not CR	44 (54.3%)	15 (78.9%)	174 (50.6%)	26 (46.4%)	
HLA-matching status, *n* (%)					0.382
Mismatch	55 (67.9%)	13 (68.4%)	246 (71.5%)	41 (73.2%)	
Match	23 (28.4%)	4 (21.1%)	64 (18.6%)	10 (17.9%)	
Unrelated donor	3 (3.7%)	2 (10.5%)	34 (9.9%)	5 (8.9%)	
MNC, median (IQR)	8.11 (6.19, 11.7)	6.72 (5.61, 9.60)	7.955 (6.4, 11.31)	7.855 (6.45, 10.54)	0.298
CD34, median (IQR)	4.04 (2.71, 7.93)	3.47 (2.67, 6.2)	4.15 (2.92, 7.46)	4.725 (2.78, 6.37)	0.712
Neutrophil engraftment, *n* (%)					**<0.001**
Failure	8 (9.9%)	5 (26.3%)	2 (0.6%)	0 (0%)	
Engraftment	73 (90.1%)	14 (73.7%)	342 (99.4%)	56 (100%)	
Neutrophil engraftment time, median (IQR)	12 (12, 16)	16 (15, 19)	13 (11, 16)	15 (12.75, 18)	**<0.001**
PLT engraftment, *n* (%)					**<0.001**
Failure	11 (13.6%)	8 (42.1%)	14 (4.1%)	7 (12.5%)	
Engraftment	70 (86.4%)	11 (57.9%)	330 (95.9%)	49 (87.5%)	
PLT engraftment time, median (IQR)	13 (12, 16.75)	21 (16, 24.5)	14 (12, 19)	17 (14, 21)	**<0.001**
aGVHD, *n* (%)					0.756
No GVHD	54 (66.7%)	13 (68.4%)	232 (67.4%)	35 (62.5%)	
Grade III–IV	12 (14.8%)	4 (21.1%)	64 (18.6%)	14 (25%)	
Grade I–II	15 (18.5%)	2 (10.5%)	48 (14%)	7 (12.5%)	
Pretreatment, *n* (%)					0.304
MAC	68 (84%)	16 (84.2%)	258 (75%)	44 (78.6%)	
RIC	13 (16%)	3 (15.8%)	86 (25%)	12 (21.4%)	
ATG dose, *n* (%)					0.044
10 mg/kg	45 (55.6%)	8 (42.1%)	121 (35.2%)	22 (39.3%)	
7.5 mg/kg	27 (33.3%)	9 (47.4%)	187 (54.4%)	29 (51.8%)	
5 mg/kg	9 (11.1%)	2 (10.5%)	36 (10.5%)	5 (8.9%)	

CMVr, Cytomegalovirus reactivation; BSI, Bloodstream infection; IQR, Interquartile range; AML, Chronic myeloid leukaemia; AA, Aplastic anaemia; AITL, Angioimmunoblastic T-cell lymphoma; Not CR, partial response, stable disease (SD), and progressive disease; CR, Complete response; MNC, Mononuclear Cell; PLT, Platelets; aGVHD, acute Graft-Versus-Host Disease; MAC, Myeloablative Conditioning; RIC, Reduced-Intensity Conditioning; ATG dose, Anti-Thymocyte Globulin dose.

Dividing all 500 patients into CMVr and No-CMVr groups, landmark analysis was conducted to assess the impact of CMVr on survival. Within the first 60 days post-transplantation, the No-CMVr group exhibited poorer survival compared to the CMVr group (Supplementary Figure 2A). Baseline analysis comparing the two groups, as shown in Table 2, revealed a significantly higher proportion of neutrophil engraftment failure patients in the No-CMVr group (*p* < 0.001). Additionally, analysis of causes of death within the first 60 days post-transplantation showed that the proportion of infection-related deaths in the No-CMVr group reached 76%, significantly higher than that in the CMVr group (Supplementary Table 3). Therefore, the atypical finding of decreased survival in the No-CMVr group within the first 60 days post-transplantation is attributed to the increased probability of pathogenic infections due to neutropenia. However, from the 60 days post-HSCT to the long-term follow up, the no-CMVr group showed a trend towards better survival than the CMVr group, although this was not significant (*p* = 0.18; Supplementary Figure 2B).

To evaluate the occurrence of combined BSI and CMVr on patient survival within the first 100 days after transplantation, we categorised patients with CMVr into two groups: (1) CMVr with BSI (*n* = 56) and (2) CMVr without BSI (*n* = 344). The characteristics are summarised in Supplementary Table 4. The 6-month OS and long-term survival were lower in the CMVr with BSI group than in the CMVr without BSI group (Supplementary Figures 3A,B).

To further exclude the influence of GVHD on the survival of that CMVr with BSI, we selected the patient without aGVHD and divided them into CMVr with BSI and CMVr without BSI. The results were consistent with those obtained previously, as illustrated in Supplementary Figure 4. The CMVr without BSI group demonstrated significantly better survival than the CMVr with BSI group (*p* = 0.0347).

### 3.5 Risk factors for BSI in patients with CMVr

After 60 days post-transplantation, there was a significant decrease in survival for CMVr with BSI patients. Subsequently, we further conducted logistic regression analysis to analyse the risk factors for BSI in patients with CMVr. Variables associated with BSI after HSCT were previously reported to include a diagnosis of acute leukaemia and MDS, transplantation from an HLA-mismatched donor and cord blood, an interval from diagnosis to HSCT of ≥190 days, carbapenem therapy, grade 3–4 intestinal mucositis, older age, and the duration of severe neutropenia (Girmenia et al., 2017; Cho et al., 2019; Ren et al., 2019). Therefore, we selected age, sex, donor origin, intensive conditioning regimen, total body irradiation, ATG dose, and aGVHD severity for logistic regression analysis. This revealed that the time to platelet engraftment (odds radio [OR] 1.007, 95% CI 1.002–1.012, *p* = 0.007) was associated with BSI after HSCT in patients with CMVr. Multivariate analysis identified the time to platelet engraftment (OR 1.007, 95% CI 1.002–1.012, *p* = 0.007) as an independent risk factor for BSI within 100 days after allo-HSCT (Table 5).

**Table 5:** Logistic regression analysis of bloodstream infection (BSI) in patients with cytomegalovirus reactivation (CMVr) within 100 days of allogeneic haematopoietic stem cell transplantation (allo-HCST).

Characteristics	Total (*N*)	Univariate analysis		Multivariate analysis	
		Odds ratio (95% CI)	*p*-value	Odds Ratio (95% CI)	*p*-value
Patient age (years)	400				
<18	107	Reference			
≥18	293	1.112 (0.462–1.762)	0.750		
Patient sex	400				
Male	251	Reference			
Female	149	1.206 (0.630–1.782)	0.524		
Disease	400				
No Acute leukaemia	119	Reference			
Acute leukaemia	281	0.967 (0.352–1.582)	0.915		
Disease status	400				
CR	201	Reference			
Not CR	199	0.857 (0.291–1.423)	0.592		
Donor sex	400				
Male	265	Reference			
Female	135	1.106 (0.516–1.697)	0.738		
Donor age(years)	400				
<18	32	Reference			
≥18	368	1.626 (0.403–2.850)	0.436		
HLA-match	400				
Unrelated donor	39	Reference			
Match	74	1.062 (−0.089–2.214)	0.918		
Mismatch	287	1.133 (0.138–2.129)	0.805		
MNC	400	0.959 (0.889–1.029)	0.238		
CD34	400	0.976 (0.907–1.046)	0.500		
Time of neutrophil engraftment	400	1.002 (0.984–1.020)	0.796		
Time of PLT engraftment	400	1.007 (1.002–1.012)	**0.007**	1.007 (1.002–1.012)	**0.007**
aGVHD	400				
No aGVHD	263	Reference			
I-II aGVHD	60	1.036 (0.209–1.863)	0.933		
III-IV aGVHD	77	1.497 (0.815–2.179)	0.246		
Pretreatment	400				
RIC	98	Reference			
MAC	302	1.222 (0.539–1.906)	0.565		
ATG dose	400				
5 mg/kg	41	Reference			
7.5 mg/kg	216	1.117 (0.103–2.130)	0.831		
10 mg/kg	143	1.309 (0.269–2.349)	0.612		

CMVr, Cytomegalovirus reactivation; BSI, Bloodstream infection; IQR, Interquartile range; Not CR, partial response, stable disease (SD), and progressive disease; CR, Complete response; MNC, Mononuclear cell; PLT, Platelets; aGVHD, acute graft-versus-host disease; MAC, Myeloablative conditioning; RIC, Reduced-intensity conditioning; ATG dose, Anti-thymocyte globulin dose.

## 4 Discussion

This study conducted a comprehensive evaluation of clinical outcomes and survival amongst a cohort of patients undergoing allo-HSCT. It assessed the impact of CMVr and BSI on patient survival at different times after transplantation and identified associated risk factors. This analysis has not yet been applied in large-scale population-based studies of allo-HSCT.

Previous studies have demonstrated a CMVr rate after HSCT ranging from approximately 30 to 70% (Schmidt-Hieber et al., 2013; Green et al., 2016; Styczynski et al., 2016; Teira et al., 2016; Maertens and Lyon, 2017; Schuster et al., 2017; Kim et al., 2020). In our study, we observed a higher incidence of CMVr (76.04%) at 100 days after allo-HSCT. This elevated rate may be attributed to several factors, including the all patients in our cohort being CMV-IgG-positive and receiving higher doses of ATG during allo-HSCT. The pre-transplant seropositivity positive of recipient is identified as an independent risk factor for post-transplant CMV reactivation. In our study, all 500 patients and their donors tested positive for serological CMV IgG before transplantation. Considering the high prevalence of CMV infection in the southeastern coastal regions of China, where this study was conducted, it is not surprising that previous studies have not indicated CMVr rates exceeding 70% (Fang et al., 2009; Zhang et al., 2014). In China, a critical scarcity of HLA-matched donors leads most HSCT patients to receive haploidentical transplants (Xu et al., 2023). Consequently, patients with HLA mismatches represented more than 70% of our study cohort, with virtually all undergoing ATG treatment to GVHD. This contrasts sharply with the CIBMTR data, where HLA-mismatched donors constitute less than 5%, and ATG usage is below 30% (Teira et al., 2016). Valganciclovir, a prodrug of ganciclovir, has a bioavailability of 60%, markedly higher than ganciclovir’s 6.3%. If this initial treatment proves ineffective, we then consider switching to valganciclovir or foscarnet (Pescovitz et al., 2000). Ganciclovir or Foscarnet preemptive therapy can effectively prevent CMV end-organ disease, but this strategy does not reduce CMV DNAemia/antigenemia and its indirect impact on Non-Relapse Mortality (NRM; de la Camara, 2016). Recent studies indicate that Letermovir prophylactic treatment can inhibit clinically significant Cytomegalovirus infection in patients from any transplant source and is significantly associated with a reduction in NRM 6 months post-bone marrow transplantation (Toya et al., 2024). Thus, from April 2023, our centre began using Letermovir for CMV prophylaxis, but the number of patients was small and not included in the cohort of this study.

Most studies indicate that the median time for CMVr falls within the range of days +30 to +60 (Teira et al., 2016; Goldsmith et al., 2021; Lin et al., 2023). In this study, the median time for CMVr was days +32. Recent research indicates that the cumulative incidence of CMV infection reaches its peak and tends to stabilise around 60 days after HSCT (Huang et al., 2024). Therefore, this study chose days +60 as the landmark. Then, the survival rate of the BSI only group significantly decreased from day 0 to day +60, indicating that BSI significantly affects patient survival within 60 days after transplantation. Therefore, before 60 days post-transplantation, the impact of BSI and CMVr on survival is not on the same scale. To analyse the impact of CMVr with BSI on survival, we believe that choosing day +60 as a landmark to analyse the impact of CMVr with BSI on survival yields more rational results. This approach can exclude the issue where the influence of BSI on overall survival significantly ‘outshines’ the CMVr variable.

We observed that patients in the CMVr group seemed to have better survival than those without CMVr in the first 60 days after transplantation. However, baseline characteristics between the two groups showed better neutrophil engraftment in the CMVr group. Hence, the variance in survival rates during the initial 60-day period following transplantation could be ascribed to heightened mortality from infections in the no-CMVr cohort, stemming from inadequate neutrophil engraftment. Such fatalities may occur before the manifestation of CMVr.

After the initial 60 days after transplantation, the OS rate for patients without CMVr was 70.9%, slightly exceeding the 63.4% observed in the CMVr group. Whilst the difference between the two groups was not statistically significant, the findings align more closely with those of previous studies indicating a negative impact of CMVr on survival in patients after allo-HSCT (Teira et al., 2016). Several factors influence CMVr after allo-HSCT, including the serum status of CMV in donors and patients, the allo-HSCT regimen, and prophylactic medications. This difference from other studies may be attributed to the increased attention and standardisation in CMV detection, prevention, and treatment by transplant doctors. Prospective, multicentre, randomised controlled studies are required for confirmation.

One of the main contributions of this study was the investigation of the impact of CMVr and BSI on survival before and after 60 days after allo-HSCT. We observed a crossover between survival curves around 60 days after transplantation between the no-infection group and CMVr-only group, indicating that this time point may represent a critical juncture for the survival of patients with CMVr. The landmark analysis further demonstrated a decline in survival for patients with CMVr with or without BSI from 60 days after transplantation to long-term follow-up. Notably, patients with CMVr combined with BSI exhibited worse survival after 60 days following transplantation. In light of the commonality of infections and aGVHD following transplantation, Poutsiaka et al. (2011) identified a correlation between BSI and aGVHD. Given that GVHD is a significant risk factor for mortality after transplantation, we conducted further analyses to ascertain if aGVHD affected the survival rates in patients with CMVr and BSI 60 days after transplantation. This separate analysis revealed that the survival rates were significantly lower in the CMVr with BSI group than in the CMVr without BSI group.

Our findings suggest that the median time for CMVr is 32 days after transplantation. In our cohort, most cases of BSI occurred before CMVr, with only five cases of BSI occurring after CMVr amongst a cohort of 500 patients. We have yet to confirm the relationship between CMVr and BSI. However, previous studies showing that CMVr can indirectly affect engraftment and immunosuppression, potentially leading to concurrent bacterial or fungal infections (Chan and Logan, 2017; Cho et al., 2019; Fan et al., 2022).

Our study had some limitations that should be acknowledged. (1) For example, the retrospective design may have introduced bias and confounding factors beyond our control. (2) The timing of CMVr and BSI events was determined based on laboratory test results. However, BSI detection, conducted using blood culture, has a lower sensitivity and longer diagnostic time. Therefore, we could not accurately determine the duration of BSI and precisely calculate the proportion of simultaneous occurrence of BSI and CMVr. (3) We did not have complete data on antibiotic and steroid use because of which it was difficult to determine the risk factors associated with BSI occurrence. (4) Due to patient compliance and financial constraints, we were unable to collect data on T-cell recovery at various stages post-transplantation for all patients. (5) Due to limitations in medical resources, we cannot conduct tissue organ biopsies to diagnose CMV end-organ disease definitively. Diagnosis relies solely on clinical assessment rather than laboratory confirmation. Consequently, complete data on CMV disease in this cohort cannot be collected. Despite these limitations, our study provides valuable insights into the impact of CMVr and BSI on survival within the context of stem cell implantation and transplantation. Future research should delve further into the mechanisms behind CMVr’s potential impact on immune function and infection risk.

In conclusion, our evaluation of CMVr and BSI following transplantation demonstrates that whilst CMVr alone does not compromise transplant prognosis, its concurrence with BSI markedly affects patient outcomes. Our findings indicate that patients afflicted with both CMVr and BSI shortly after transplantation face a significantly reduced survival rate beyond 60 days. In the initial post-transplant phase (within 60 days), the risk of mortality is heightened by BSIs, yet those who overcome BSIs during this period exhibit a decreased mortality risk thereafter. Nevertheless, the presence of CMVr in patients with BSI considerably diminishes their long-term survival prospects, underscoring the vital need for strategic preventive and early management measures targeting CMVr and BSIs. Therefore, in the clinical setting, it’s importance to focus on BSI monitoring in the first 60 days following transplantation and subsequently, to focus on the detection and treatment of CMVr to enhance patient outcomes amongst allo-HSCT recipients.

## Data availability statement

Original data have been deposited at Zenodo and are publicly available as of the date of publication https://doi.org/10.5281/zenodo.10720246.

## Ethics statement

The research was conducted by the ethical standards of the local institutional review board (the Medical Ethics Committee of Fujian Medical University Union Hospital, 2022KY167). The studies were conducted in accordance with the local legislation and institutional requirements. Written informed consent for participation in this study was provided by the participants’ legal guardians/next of kin.

## Author contributions

JR: Conceptualization, Data curation, Formal analysis, Funding acquisition, Methodology, Project administration, Software, Writing – original draft. JX: Conceptualization, Data curation, Formal analysis, Funding acquisition, Methodology, Project administration, Software, Writing – original draft, Writing – review & editing. JS: Data curation, Investigation, Methodology, Writing – review & editing. XW: Data curation, Investigation, Writing – review & editing. XY: Data curation, Investigation, Writing – review & editing. CN: Data curation, Resources, Writing – review & editing. LL: Data curation, Resources, Writing – review & editing. YZ: Data curation, Resources, Writing – review & editing. XZ: Data curation, Methodology, Writing – review & editing. JL: Data curation, Resources, Writing – review & editing. QL: Data curation, Funding acquisition, Resources, Writing – review & editing. JH: Project administration, Resources, Supervision, Writing – review & editing. TY: Conceptualization, Project administration, Resources, Supervision, Writing – review & editing.

## Supplementary material

SUPPLEMENTARY FIGURE 1 **(A)** Landmark analysis of overall survival for all patients within 180 days after allogeneic haematopoietic stem cell transplantation (allo-HCST); landmark point set at 30 days after transplantation; before 30 days, *p* = 0.513; after 30 days, *p* < 0.001; **(B)** Landmark analysis of overall survival for all patients after allo-HSCT patients in long-term follow-up; landmark point set at 30 days after transplantation; before 30 days, *p* = 0.513; after 30 days, *p* = 0.001.

SUPPLEMENTARY FIGURE 2 **(A)** Landmark analysis of overall survival for the cytomegalovirus reactivation (CMVr) and no-CMVr groups within 180 days after transplantation; landmark point set at 60 days after transplantation; the p-values for both groups before and after 60 days are 0.002 and 0.163, respectively; **(B)** Landmark analysis of overall survival for the CMVr and no-CMVr groups in long-term follow-up; landmark point set at 60 days after transplantation; the p-values for both groups before and after 60 days are 0.002 and 0.18, respectively.

SUPPLEMENTARY FIGURE 3 **(A)** The 180-day OS was lower in the cytomegalovirus reactivation (CMVr) with bloodstream infection (BSI) group than in the CMVr with no BSI group (58.9% vs. 78.9%, *p* = 0.0003, HR 3.244 [95% CI 1.763–5.968]); **(B)** Long-term survival was lower in the CMVr with BSI group than in the CMVr with no BSI group (58.9% vs. 79.6%, *p* = 0.0002, HR 3.131 [95% CI 1.688–5.808]).

SUPPLEMENTARY FIGURE 4 Kaplan–Meier analysis of long-term survival in patients without acute GVHD (aGVHD).
