# Supplementary figures and images for: Reactivation of cytomegalovirus and bloodstream infection and its impact on early survival after allogeneic haematopoietic stem cell transplantation: a multicentre retrospective study
Source: Front Microbiol. 2024 Jun 19;15:1405652. doi: 10.3389/fmicb.2024.1405652 (PMC11219566; doi:10.3389/fmicb.2024.1405652)

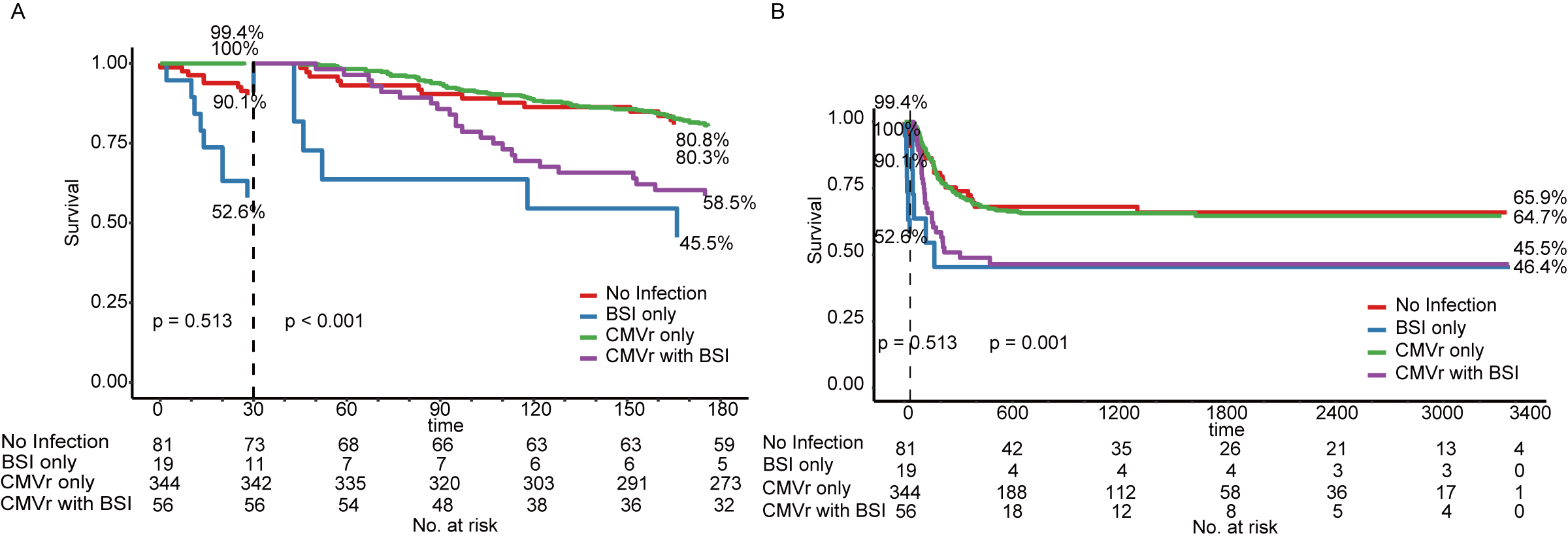

Supplement: SUPPLEMENTARY FIGURE 1 — (A) Landmark analysis of overall survival for all patients within 180 days after allogeneic haematopoietic stem cell transplantation (allo-HCST); landmark point set at 30 days after transplantation; before 30 days, p = 0.513; after 30 days, p < 0.001; (B) Landmark analysis of overall survival for all patients after allo-HSCT patients in long-term follow-up; landmark point set at 30 days after transplantation; before 30 days, p = 0.513; after 30 days, p = 0.001. [file Image_1.TIF]

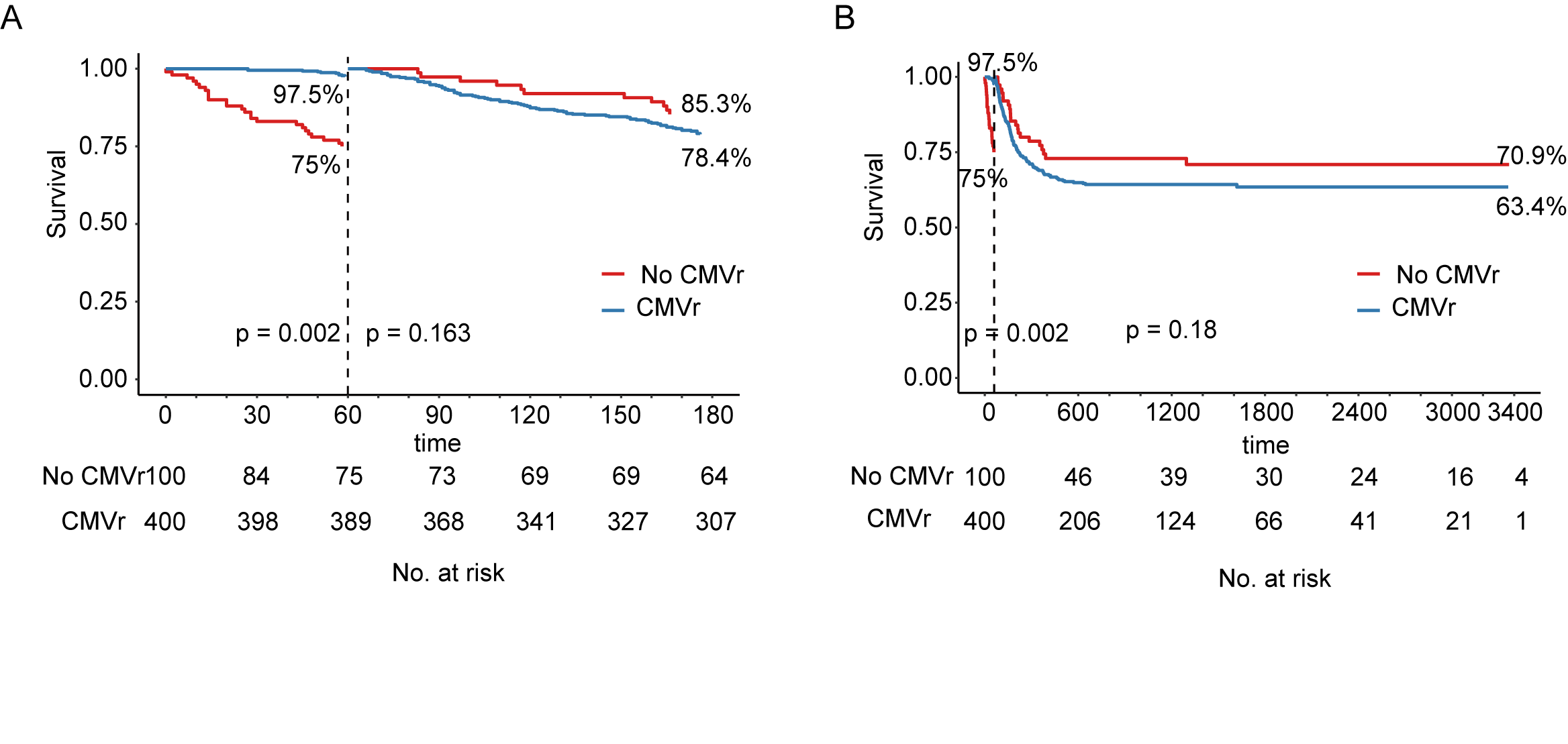

Supplement: SUPPLEMENTARY FIGURE 2 — (A) Landmark analysis of overall survival for the cytomegalovirus reactivation (CMVr) and no-CMVr groups within 180 days after transplantation; landmark point set at 60 days after transplantation; the p-values for both groups before and after 60 days are 0.002 and 0.163, respectively; (B) Landmark analysis of overall survival for the CMVr and no-CMVr groups in long-term follow-up; landmark point set at 60 days after transplantation; the p-values for both groups before and after 60 days are 0.002 and 0.18, respectively. [file Image_2.TIF]

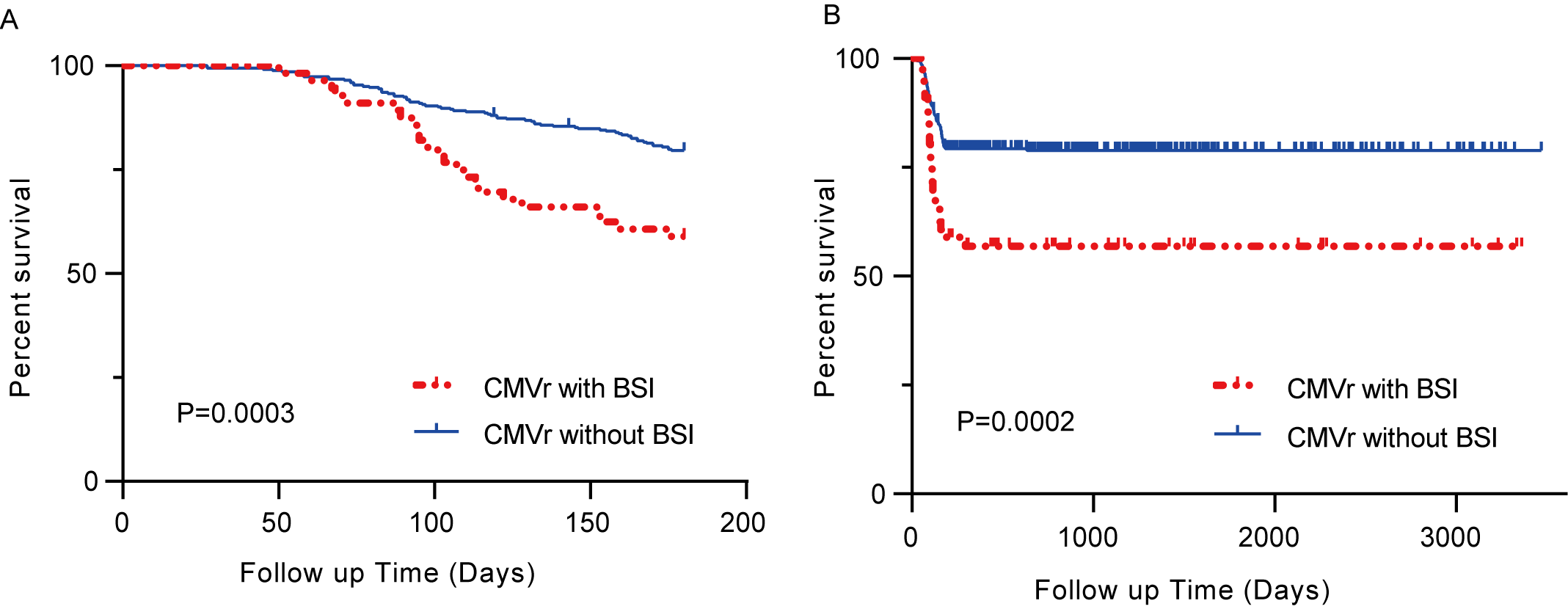

Supplement: SUPPLEMENTARY FIGURE 3 — (A) The 180-day OS was lower in the cytomegalovirus reactivation (CMVr) with bloodstream infection (BSI) group than in the CMVr with no BSI group (58.9% vs. 78.9%, p = 0.0003, HR 3.244 [95% CI 1.763–5.968]); (B) Long-term survival was lower in the CMVr with BSI group than in the CMVr with no BSI group (58.9% vs. 79.6%, p = 0.0002, HR 3.131 [95% CI 1.688–5.808]). [file Image_3.TIF]

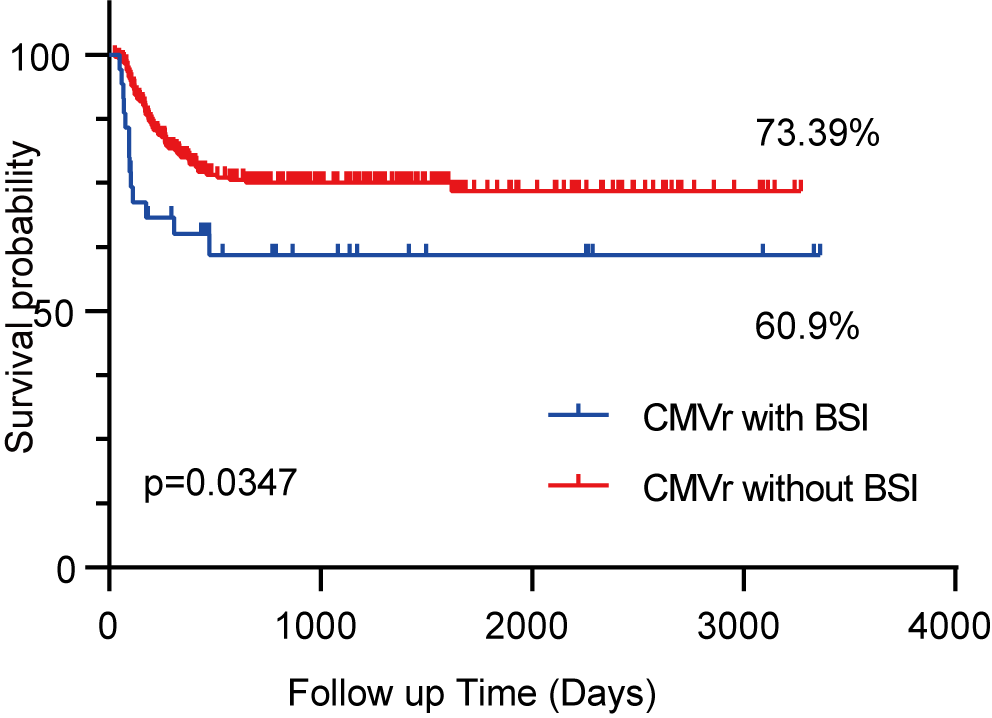

Supplement: SUPPLEMENTARY FIGURE 4 — Kaplan–Meier analysis of long-term survival in patients without acute GVHD (aGVHD). [file Image_4.TIF]
