# Supplementary material for: Reactivation of cytomegalovirus and bloodstream infection and its impact on early survival after allogeneic haematopoietic stem cell transplantation: a multicentre retrospective study
Source: Front Microbiol. 2024 Jun 19;15:1405652. doi: 10.3389/fmicb.2024.1405652 (PMC11219566; doi:10.3389/fmicb.2024.1405652)
Supplement: Supplementary file 5 [file Data_Sheet_1.pdf]

Supplementary Table 1. Causative Organisms Isolated from BSI Blood Samples of the allo-HSCT

Characteristics	Total（n=75）
GNB, n (%)	42(56.0%)
Klebsiella pneumoniae, n (%)	22 (52.3%)
Escherichia coli, n (%)	20(47.6%)
Pseudomonas aeruginosa, n (%)	14 (33.3%)
Stenotrophomonas maltophilia, n (%)	4 (9.5%)
Pseudomonas putida, n (%)	2 (4.8%)
GPB, n (%)	26(34.7%)
Staphylococci, n (%)	10 (38.5%)
Streptococcus, n (%)	12 (46.1%)
Enterococcus faecium, n (%)	4 (15.4%)
Fungus, n (%)	6 (23.1%)
others, n (%)	3 (4.0%)

Abbreviations: GNB: gram-negative bacteria; GPB: gram-positive bacteria.

Supplementary Table 2. Rates of multidrug resistance among the major taxa of pathogens detected from BSIs of the allo-HSCT.

**Multidrug-resistance bacterial**	**Total（n=75）**
MDR, n (%)	35(46.7%)
MRSA, n (%)	3 (4.0%)
ESBL(+), n (%)	16(21.3%)
CRE, n (%)	5 (6.7%)
CRPA, n (%)	11 (14.7%)
No MDR, n (%)	40(53.3%)

Abbreviations: MDR: Multidrug resistance; MRSA: Methicillin-resistant Staphylococcus aureus; ESBL (+): Extended spectrum β-lactamase-producing; CRE: Carbapenem-resistant Enterobacterium; CRPA: Carbapenem-resistant Pseudomonas aeruginosa.

Supplementary Table 3. The causes of death in the cytomegalovirus reactivation (CMVr) group and no-CMVr group during the first 60 days after transplantation

Characteristics	No CMVr<br>N=25	CMVr<br>N=11
Infection, n (%)	19 （76%）	4（36%）
Relapse, n (%)	0（0%）	1（9.09%）
AGVHD, n (%)	1（4%）	5（45.45%）
Bleeding, n (%)	1（4%）	1（9.09%）
Others, n (%)	3（12%）	2（18.18%）

Abbreviations: CMVr: Cytomegalovirus Reactivation; AGVHD: acute Graft-Versus-Host Diseas.

Supplementary Table 4. Baseline characteristics of all patients in the cytomegalovirus reactivation (CMVr) and no-CMVr groups with/without bloodstream infection (BSI)

Characteristics	No CMVr<br>N=100	CMVr with BSI<br>N=56	CMVr without BSI<br>N=344	P value
**Age, median (IQR)**	25.5 (11, 39.25)	25 (17.75, 36.25)	26 (12, 40.25)	0.921
**Patient Sex, n (%)**				0.191
Male	72 (72%)	33 (58.9%)	219 (63.7%)	
Female	28 (28%)	23 (41.1%)	125 (36.3%)	
**Disease, n (%)**				0.222
AML	42 (42%)	23 (41.1%)	143 (41.6%)	
ALL	21 (21%)	14 (25%)	80 (23.3%)	
MDS	9 (9%)	4 (7.1%)	19 (5.5%)	
CML	10 (10%)	2 (3.6%)	13 (3.8%)	
AA	15 (15%)	7 (12.5%)	57 (16.6%)	
AITL	2 (2%)	0 (0%)	5 (1.5%)	
lymphoma	0(0%)	4 (7.1%)	13 (3.8%)	
Other	1(1%)	2(3.6%)	14 (4.1%)	
**Disease Status, n (%)**				0.161
Not CR	60 (60%)	26 (46.4%)	173 (50.3%)	
CR	40 (40%)	30 (53.6%)	171 (49.7%)	

Characteristics	No CMVr<br>N=100	CMVr with BSI<br>N=56	CMVr without BSI<br>N=344	P value
**Donor Sex, n (%)**				0.275
Male	58 (58%)	35 (62.5%)	229 (66.6%)	
Female	42 (42%)	21 (37.5%)	115 (33.4%)	
Donor Age, mean ± sd	33.98 ± 11.674	34.339 ± 11.1	33.936 ± 11.531	0.971
**HLA-matching Status, n (%)**				0.272
Match	27 (27%)	10 (17.9%)	64 (18.6%)	
Mismatch	68(68%)	41 (73.2%)	246 (71.5%)	
Unrelated donor	5 (5%)	5 (8.9%)	34 (9.9%)	
**MNC, median (IQR)**	7.8 (5.98, 11.592)	7.855 (6.4525, 10.543)	7.955 (6.4, 11.308)	0.805
**CD34, median (IQR)**	3.75 (2.7, 7.795)	4.725 (2.7825, 6.3725)	4.15 (2.915, 7.455)	0.845
**Neutrophil Engraftment, n (%)**				< 0.001
Engraftment	87 (87%)	56 (100%)	342 (99.4%)	
Failure	13 (13%)	0 (0%)	2 (0.6%)	
**PLT engraftment, n (%)**				< 0.001
Engraftment	81 (81%)	49 (87.5%)	330 (95.9%)	
Failure	19 (19%)	7 (12.5%)	14 (4.1%)	
**aGVHD, n (%)**				0.662
No aGVHD	67 (67%)	35 (62.5%)	232 (67.4%)	
Grade I-II	17 (17%)	7 (12.5%)	48 (14%)	
Grade III-IV	16 (16%)	14 (25%)	64 (18.6%)	
**Pretreatment, n (%)**				0.163
RIC	16 (16%)	12 (21.4%)	86 (25%)	
MAC	84 (84%)	44 (78.6%)	258 (75%)	
**ATG dose, n (%)**				0.02
5 mg/kg	11 (11%)	5 (8.9%)	36 (10.5%)	
7.5 mg/kg	36 (36%)	29(51.8%)	187(54.4%)	
10 mg/kg	53(53%)	22(39.3%)	121(35.2%)	
**BSI, n (%)**				< 0.001

Characteristics	No CMVr<br>N=100	CMVr with BSI<br>N=56	CMVr without BSI<br>N=344	P value
No BSI	81 (81%)	0 (0%)	344 (100%)	
BSI	19 (19%)	56 (100%)	0 (0%)	
**CMVr Time (day), mean ± sd**		37.26 ± 19.1	33.5 ± 16.2	0.289
**Duration of CMVr (day), mean ± sd**		35 (22.5, 48.75)	35 (16, 55.5)	0.166
**Follow-up time (day), median (IQR)**	384 (76.75, 2068.5)	298.5 (109.25, 1145.8)	673.5 (225.5, 1394.5)	0.035

Abbreviations: CMVr: Cytomegalovirus Reactivation; BSI: Bloodstream Infection; IQR: Interquartile Range; AML: Acute Myeloid Leukemia; ALL: Acute Lymphoblastic Leukemia; MDS: Myelodysplastic Syndromes; CML: Chronic Myeloid Leukemia; AA: Aplastic Anemia; AITL: Angioimmunoblastic T-cell Lymphoma; Not CR: partial response, stable disease (SD), and progressive disease; CR: Complete Response; MNC: Mononuclear Cell; PLT: Platelets; aGVHD: acute Graft-Versus-Host Disease; MAC: Myeloablative Conditioning; RIC: Reduced-Intensity Conditioning; ATG dose: Anti-Thymocyte Globulin dose.
